# Lifestyle-related disease in an emergency department setting

**DOI:** 10.1186/1757-7241-23-S1-A34

**Published:** 2015-07-16

**Authors:** Emilie Bækgaard, Thomas A Schmidt

**Affiliations:** 1 The Emergency Department, Holbaek Hospital, Copenhagen University Hospital, Copenhagen, Denmark

## Background

According to the WHO, non-communicable (non-infectious) diseases account for 90% of all deaths in Denmark. The development of these diseases can often be contributed to lifestyle factors, namely diet, smoking, and alcohol, and, in association with these, type 2 diabetes, hypertension, and hypercholesterolaemia. One could argue that much of the burden of non-communicable disease actually is **preventable **disease, and, as such, could be eliminated through modification of lifestyle elements. The purpose of this project was to map the incidence of admissions attributable to lifestyle-related diseases, during November and December 2012 at a Danish Acute Admissions Department.

## Methods

Every patient contact is given a diagnostic code upon leaving (at discharge or transfer elsewhere). A pre-determined list of diagnostic codes, covering admissions related to alcohol, chronic obstructive pulmonary disease, ischaemic heart disease, cerebrovascular disease, type II diabetes, and recreational drug use, was created. Patient statistics were acquired from the departments' statistical database, with permission from the Danish Data Protection Agency. Furthermore, medical records for all patients admitted to the hospital during a randomly selected 24-hour period (within the study time) were examined manually, in order to identify lifestyle-related elements as risk factors, and, indeed, complicating factors, for disease. These were analysed to produce an overview of 'lifestyle-related' patient contacts.

## Results

6,699 patient contacts were analysed. 320 patients were admitted purely on the basis of a lifestyle-related disease, corresponding to one patient every 4-6 hours, 24 hours a day, 7 days a week.

During the 24-hour period, however, 14 patients (out of 89 admitted, 12.5%) fit the criteria - corresponding to one patient every 102 minutes. Furthermore, 67% of patients seen in those 24 hours had one or more lifestyle-related risk factors.

## Conclusions

Lifestyle-related diseases account for the majority of deaths in Denmark, and constitute an important source of admissions too. Over two-thirds of patients admitted through this department have at least one lifestyle-related comorbidity or risk factor; and 12.5% of admissions are potentially preventable.

